# Assessment of N/L ratio and subclinical atherosclerosis in FH subjects with or without LDLR mutation

**DOI:** 10.1210/jendso/bvag001

**Published:** 2026-01-08

**Authors:** Francesco Di Giacomo Barbagallo, Giosiana Bosco, Maurizio Di Marco, Sabrina Scilletta, Nicoletta Miano, Marina Martedì, Ivan Privitera, Maria Chiara Papa, Chiara Piazza, Francesca Valenza, Giovanni Pennisi, Ernestina Marianna De Francesco, Roberta Malaguarnera, Antonino Di Pino, Salvatore Piro, Roberto Scicali

**Affiliations:** Department of Clinical and Experimental Medicine, University of Catania, Catania 95123, Italy; Department of Medicine and Surgery, “Kore” University of Enna, Enna 94100, Italy; Department of Clinical and Experimental Medicine, University of Catania, Catania 95123, Italy; Department of Medicine and Surgery, “Kore” University of Enna, Enna 94100, Italy; Department of Clinical and Experimental Medicine, University of Catania, Catania 95123, Italy; Department of Clinical and Experimental Medicine, University of Catania, Catania 95123, Italy; Department of Clinical and Experimental Medicine, University of Catania, Catania 95123, Italy; Department of Clinical and Experimental Medicine, University of Catania, Catania 95123, Italy; Department of Clinical and Experimental Medicine, University of Catania, Catania 95123, Italy; Department of Clinical and Experimental Medicine, University of Catania, Catania 95123, Italy; Department of Clinical and Experimental Medicine, University of Catania, Catania 95123, Italy; Department of Clinical and Experimental Medicine, University of Catania, Catania 95123, Italy; Department of Clinical and Experimental Medicine, University of Catania, Catania 95123, Italy; Department of Medicine and Surgery, “Kore” University of Enna, Enna 94100, Italy; Department of Medicine and Surgery, “Kore” University of Enna, Enna 94100, Italy; Department of Clinical and Experimental Medicine, University of Catania, Catania 95123, Italy; Department of Clinical and Experimental Medicine, University of Catania, Catania 95123, Italy; Department of Clinical and Experimental Medicine, University of Catania, Catania 95123, Italy

**Keywords:** familial hypercholesterolemia, neutrophil-to-lymphocyte ratio, LDL receptor, inflammation, subclinical atherosclerosis, cardiovascular risk

## Abstract

**Background:**

Familial hypercholesterolemia (FH) is a genetic disorder characterized by elevated low-density lipoprotein-cholesterol (LDL-C) and increased cardiovascular risk. While the role of LDL-C in atherogenesis is well established, the contribution of inflammatory activation in FH, particularly in relation to genotype, remains poorly defined. We aimed to evaluate the impact of genotype on neutrophil-to-lymphocyte ratio (NLR) and on subclinical atherosclerosis in a cohort of FH subjects.

**Methods:**

We conducted a cross-sectional study on 423 FH subjects not on lipid-lowering therapy and free from atherosclerotic cardiovascular disease. Biochemical, genetic, and vascular assessments were performed in all participants. The population was divided into 2 groups based on genotype: low-density lipoprotein receptor (LDLR; n = 273) and non-LDLR (NLDLR, n = 150). Vascular profile was assessed by coronary artery calcium score and carotid/femoral plaque presence. NLR was calculated from peripheral blood counts.

**Results:**

The LDLR group exhibited an higher NLR (2.27 ± 0.86 vs 2.05 ± 0.68, *P* < .05) than the NLDLR group. LDL-C levels and LDLR genotype were significantly associated with NLR (both *P* < .05). Multiterritorial plaque involvement was more frequent in the LDLR group than the NLDLR group (*P* for trend <.05). Age (*P* < .001), LDL-C (*P* < .001), smoking status (*P* < .05), and NLR (*P* < .05) were independently associated with subclinical atherosclerosis.

**Conclusion:**

FH subjects with LDLR mutations had a higher NLR and a more severe atherosclerosis distribution. Our findings support the role of NLR as a noninvasive biomarker of early immune activation and highlights the importance of lipoinflammatory status evaluation in FH subjects.

RESEARCH INSIGHTS **What is currently known about this topic?**

Familial hypercholesterolemia (FH) is characterized by elevated low-density lipoprotein-cholesterol levels and premature atherosclerosis. Beyond lipid accumulation, recent evidence highlights an inflammatory component contributing to vascular injury. The neutrophil-to-lymphocyte ratio (NLR) has emerged as a biomarker of systemic inflammation and cardiovascular risk in several populations, but its role in FH remains poorly defined.

**What is the key research question?**

Does the presence of an low-density lipoprotein receptor (LDLR) mutation influence the NLR profile and the extent of subclinical atherosclerosis in subjects with FH?

**What is new?**

FH subjects with LDLR mutations showed higher NLR values neutrophil and monocyte counts compared to those without LDLR mutations. Moreover, LDLR mutation carriers exhibited a greater prevalence of multiterritorial atherosclerosis, supporting a genetic-inflammatory interplay in FH.

**How might this study influence clinical practice?**

Integrating NLR evaluation into the clinical assessment of FH may help identify patients with an enhanced inflammatory phenotype and higher atherosclerotic burden, improving risk stratification beyond low-density lipoprotein-cholesterol levels.

## Background

Familial hypercholesterolemia (FH) is the most frequent monogenic disorder characterized by increased low-density lipoprotein cholesterol (LDL-C) levels and is strongly associated with atherosclerotic cardiovascular disease (ASCVD) (1). In FH subjects, different mutations in genes encoding key proteins involved in LDL-C metabolism lead to reduced LDL-C clearance and, consequently, to elevated plasma LDL-C levels (2). Among these, the majority are present in the LDL receptor (LDLR) gene, which accounts for approximately 85% to 90% of genetically confirmed cases (3), whereas a smaller proportion of variants has been described in apolipoprotein B (ApoB), proprotein convertase subtilisin/kexin type 9 (PCSK9), or apolipoprotein E (4). Notably, subjects with LDLR mutations exhibit more severe lipid profiles and a higher burden of vascular damage, including increased coronary artery calcium (CAC) compared to non-LDLR carriers (5, 6).

Although the causal role of LDL-C in atherosclerotic injury was largely evaluated, it was recently emphasized as the contribution of inflammatory pathways in modulating cardiovascular risk (7). Several studies have demonstrated the important role of inflammation in the pathophysiology of ASCVD, and it appears to be the final expression of the systemic interplay between hypercholesterolemia and the immune system during the atherosclerosis progression (8, 9).

The neutrophil-to-lymphocyte ratio (NLR) has emerged as a simple and reproducible biomarker of systemic inflammation (10). An increased NLR has been associated with the atherosclerotic burden, plaque vulnerability, and adverse cardiovascular outcomes in both primary and secondary prevention cohorts (11). In subjects with metabolic disorders, NLR correlated with coronary calcifications, arterial stiffness, and subclinical plaque formation (12).

In the past few years, the implementation novel genetic diagnostic strategies such as next-generation sequencing has improved the ability to detect FH by a comprehensive analysis of all genes implicated in the lipid disorder, including those associated with a polygenic condition (13). No data are available on the role of NLR in genotyped FH, and no studies have yet addressed its relationship with vascular injury in these subjects.

In this study, we aimed to evaluate the impact of genotype on NLR and on subclinical atherosclerosis (SA) in a cohort of FH subjects.

## Methods

### Study design and population

This was a cross-sectional observational study involving individuals with a probable or definite clinical diagnosis of FH, based on the Dutch Lipid Clinic Network criteria (score ≥6), who had undergone genetic testing from July 2016 to November 2024 (14). All participants were enrolled at the University Hospital of Catania, Italy, which is a tertiary center for the screening, diagnosis, and management of familial dyslipidemias. All participants were aged between 18 and 70 years and were not on lipid-lowering therapy at the time of enrollment. Subjects with a personal history of ASCVD, acute infections, chronic inflammatory conditions, hematological disorders, malignancies, or ongoing immunosuppressive or glucocorticoid therapy within the previous 3 months were excluded.

After a 12-hour fast, all participants underwent a physical examination and review of their clinical history as well as biochemical analyses and vascular profile evaluation by assessments of CAC score and carotid and femoral plaques. Monogenic FH was defined as the presence of a genetic variant in one of the following genes: LDLR, ApoB, PCSK9, or apolipoprotein E, whereas the diagnosis of recessive hypercholesterolemia was confirmed by the presence of 2 genetic variants in low-density lipoprotein receptor adaptor protein 1 (15). In subjects without a monogenic variant, polygenic FH was defined as the presence of a polygenic LDL-C score >0.73 (16). Subclinical atherosclerosis was defined as a CAC score >0 and/or presence of carotid and/or femoral plaques (17).

The genotype distribution observed in our cohort (88.6% LDLR variants among monogenic FH) is fully consistent with large multicenter Italian data from the LIPIGEN registry, where LDLR mutations represent 85% to 90% of genetically confirmed FH cases (14).

Arterial hypertension was defined as brachial blood pressure ≥ 140 mm Hg (systolic) and/or 90 mm Hg (diastolic) on at least 2 different occasions, or if the subject was on antihypertensive therapy (18). Body weight and height were measured, and body mass index (BMI) was calculated as weight divided by the squared value of height (kg/m²) (19). Type 2 diabetes was defined as a fasting plasma glucose ≥ 126 mg/dL on 2 consecutive readings and/or glycated hemoglobin ≥ 6.5% or the use of antidiabetic medications (20).

Smoking habits were divided into either current smoking (defined as a minimum of 1 cigarette in the last month) or not (21). Participants were stratified into 2 groups according to genotype: FH subjects with LDLR mutation (LDLR group, 273 subjects) and FH subjects without LDL receptor mutation (NLDLR group, 150 subjects).

The study was approved by the local ethics committee in accordance with the ethical standards of the institutional and national research committees and with the 1964 Declaration of Helsinki and its later amendments or comparable ethical standards. Informed consent was obtained from each subject enrolled in the study.

### Biochemical analyses

Serum total cholesterol, triglycerides (TG), high-density lipoprotein-cholesterol (HDL-C), and high-sensitivity C-reactive protein were determined using standardized enzymatic colorimetric assays (22). LDL-C was calculated according to the Friedewald formula. Non-HDL-C was derived from baseline lipid values. ApoB and apolipoprotein A1 were measured by nephelometric assay (Siemens AG Healthcare Sector, Erlangen, Germany). Lipoprotein A concentrations were determined using a latex agglutination immunoassay (23). White blood cell count (WBCC) as well as neutrophil count (NC), monocyte count (MC), and lymphocyte count (LC) were performed by a blood cell analyzer (UniCel DxH-900, Beckman Coulter, Milan, Italy) (24). The NLR was calculated as the ratio between NC and LC (25, 26). All biochemical parameters, including NLR and lipid profile, were obtained from the same fasting blood sample collected at baseline.

### CAC assessment

Each patient underwent a multidetector computed tomography (CT) scan (Definition Flash, Siemens, Erlangen, Germany), with a total radiation exposure ranging from 1 to 3 mSv. CAC was assessed using the Agatston scoring method (27). Coronary imaging was performed without contrast, using the high-resolution mode of the ultrafast CT scanner with a scan time of 100 ms, a slice thickness of 3 mm, electrocardiogram gating, and breath-hold technique. A total of 20 contiguous slices (covering 60 mm) were acquired, starting from the lower margin of the pulmonary artery bifurcation.

The presence and extent of coronary calcification were evaluated at each slice level. A calcified lesion was defined as having a CT density of at least 130 Hounsfield units and a minimum area of 1 mm². The CAC score was calculated as the product of the calcified plaque area and the maximum lesion density, classified from 1 to 4 based on Hounsfield units. All CT scans were analyzed in a specialized central reading facility and reviewed by a senior cardiovascular radiologist blinded to the patients' medical history. CAC presence was defined as a score greater than 0.

### Carotid and femoral plaque assessments

Ultrasound assessments of the carotid and femoral arteries were performed using an ACUSON Sequoia system with an 8-MHz transducer, in accordance with previously established protocols (28). Carotid evaluations involved a 1-cm segment of the distal common carotid artery, located 1 cm proximal to the carotid bulb dilation, and a 1-cm segment of the carotid bifurcation, situated 1 cm proximal to the flow divider. Similarly, femoral artery measurements were carried out on the common femoral artery, specifically 1 cm proximal to its bifurcation.

For both the right and left carotid and femoral arteries, three longitudinal sections were acquired. The presence of plaques in the carotid and femoral arteries was defined by an intima-media thickness greater than 1.5 mm. Peripheral atherosclerosis was defined as the presence of carotid and/or femoral plaques. All ultrasound examinations were conducted by a single operator blinded to patient characteristics.

### Statistical analysis

The distribution of continuous variables was assessed using the Kolmogorov-Smirnov test. Continuous variables are presented as mean ± SD for normally distributed data or median (interquartile range) for skewed data; categorical variables are expressed as frequency and percentage. Logarithmic transformation was applied to nonnormally distributed variables such as TG and high-sensitivity C-reactive protein for statistical testing. Comparisons between groups were performed using Student *t*-test or Mann-Whitney *U* test for continuous variables and χ² test for categorical variables. Atherosclerosis extension across the coronary, carotid, and femoral territories was analyzed by stratifying the study population according to the number of involved vascular districts: no territory with plaque (0-TWP), 1 (1-TWP), 2 (2-TWP), and 3 (3-TWP).

Simple linear regression analysis was used to explore associations between NLR and clinical parameters including age, sex, BMI, systolic blood pressure, smoking status, and LDL-C levels. Multivariate logistic regression analysis was conducted to assess the independent association between NLR and subclinical atherosclerosis, adjusting for clinical parameters. The variance inflation factor was estimated and found to be <2 for all covariates included.

All statistical analyses were performed using IBM SPSS Statistics for Windows version 23. For all tests, a *P* < .05 was considered significant.

## Results

**Figure 1:**
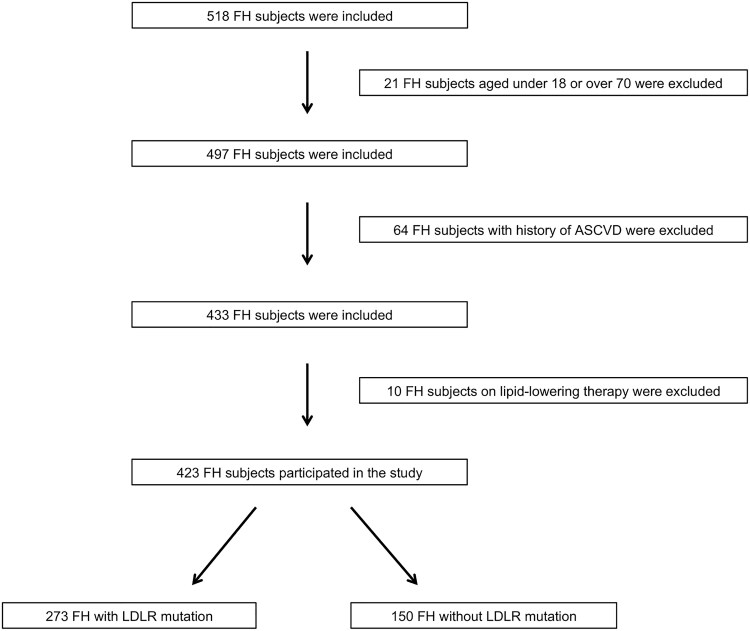
Enrollment flowchart of the study population. ASCVD, atherosclerotic cardiovascular disease; FH, familial hypercholesterolemia; LDLR, low-density lipoprotein receptor.

As reported in Fig. 1, 423 of the 518 individuals met the inclusion criteria. Subjects with history of ASCVD, lipid-lowering therapy were excluded. The study population was free from autoimmune disease as well as hormonal diseases or hormonal treatments.

The characteristics of the study population are presented in Table 1. Monogenic FH was present in 73.0% of subjects and the LDLR mutation was the most common genetic variant, whereas 27.2% of subjects exhibited a polygenic disorder. The prevalence of subclinical atherosclerosis (SA) in the study population was 62.3%; of these, CAC and peripheral plaque presences were 75.2% and 94.2%, respectively. Furthermore, the coexistence of CAC and peripheral plaque was observed in 37.3% of subjects.

**Table 1:** Characteristics of the study population

N	423
**FH genotype**	
Monogenic FH, n (%)	308 (73.0)
LDLR, n (%)	273 (88.6)
LDLR defective, n (%)	172 (63)
LDLR null, n (%)	106 (39)
ApoB, n (%)	26 (8.6)
PCSK9, n (%)	5 (1.6)
ApoE, n (%)	4 (1.5)
Polygenic FH, n (%)	115 (27.2)
**Monogenic FH phenotype**	
Heterozygous, n (%)	308 (100)
**Vascular profile**	
Subclinical atherosclerosis, n (%)	263 (62.3)
CAC > 0, n (%)	198 (75.2)
Peripheral plaque, n (%)	248 (94.2)
CAC + Peripheral plaque, n (%)	98 (37.3)

Data are presented as percentages, mean ± standard deviation (SD), or median (interquartile range), as appropriate.

Abbreviations: ApoB, apolipoprotein B; ApoE, apolipoprotein E; CAC, coronary artery calcium; FH, familial hypercholesterolemia; LDLR, low-density lipoprotein receptor; PCSK9, proprotein convertase subtilisin/kexin type 9.

Table 2 shows the general characteristics of the study population stratified into 2 groups according to the genotype. Age, BMI, and sex distribution were similar between the 2 groups. Total cholesterol (Δ +12%), LDL-C (Δ +20%), and non-HDL-C (Δ +22%) were significantly higher in the LDLR group (*P* value for all <.001), whereas TG was significantly lower in the LDLR group compared with the NLDLR group (Δ −20%; *P* < .001).

**Table 2:** Characteristics of the study population stratified according to genotype

	LDLR group	NLDLR group	*P* value between 2 groups
**Demographic characteristics**			
N	273	150	—
Age, years	55.3 ± 8.03	56.0 ± 7.61	.15
Men, n (%)	148 (54.2)	71 (47.3)	.20
Body mass index, kg/m^2^	25.7 ± 3.52	25.9 ± 3.63	.55
**Lipid profile**			
TC, mg/dL	368.0 ± 23.12	327.0 ± 21.62	<.001
HDL-C, mg/dL	52 .1 ± 6.51	54.3 ± 8.23	.11
Triglycerides, mg/dL	83 (65-106)	104 (78-142)	<.001
LDL-C, mg/dL	271.12 ± 25.03	226.07 ± 24.73	<.001
Non-HDL-C, mg/dL	157.93 ± 30.17	129.02 ± 25.64	<.001
ApoB, mg/dL	140.2 ± 5.14	137.9 ± 5.23	.79
ApoA1, mg/dL	137.5 ± 10.8	141.1 ± 10.55	.19
ApoB to ApoA1 ratio	1.05 ± 0.43	0.96 ± 0.25	.17
Lp(a), mg/dL	21.0 (10.5-41.0)	20.1 (10.8-58.0)	.13
**Risk factors**	119.2 ± 9.61		.45
Systolic BP, mm Hg		120.0 ± 9.37	
Diastolic BP, mm Hg	70.71 ± 9.17	72.82 ± 9.94	.12
Smoking, n (%)	92 (33.7)	51 (34)	.85
**Inflammatory profile**			
hs-CRP, mg/dL	0.13 (0.05-0.26)	0.10 (0.05-0.22)	.21

Data are presented as mean ± SD, median (interquartile range), or percentages.

Abbreviations: ApoA1, apolipoprotein A1; ApoB, apolipoprotein B; BP, blood pressure; FPG, fasting plasma glucose; HbA1c, glycated hemoglobin; HDL-C, high-density lipoprotein cholesterol; hs-CRP, high-sensitivity C-reactive protein; LDL-C, low-density lipoprotein cholesterol; LDLR, low-density lipoprotein receptor; Lp(a), lipoprotein(a); NLDLR, non-low-density lipoprotein receptor; non-HDL-C, non-high-density lipoprotein cholesterol; TC, total cholesterol; TG, triglycerides; TyG, triglyceride-glucose index.

**Figure 2:**
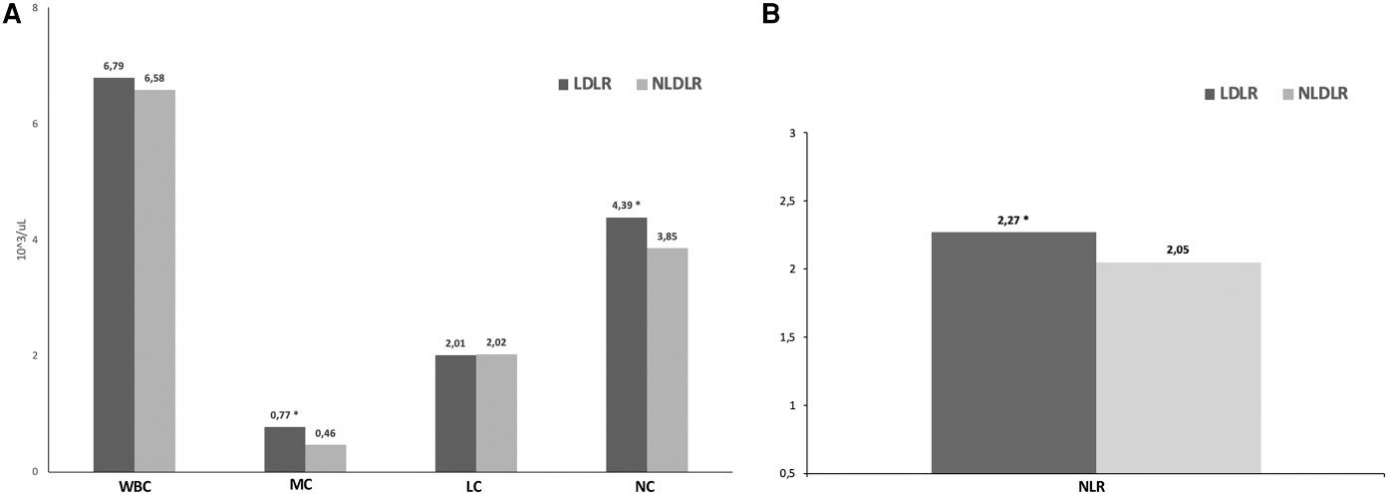
Distribution of innate immune profile and neutrophil-to-lymphocyte ratio (NLR) in the study population stratified according to genotype. (A) Distribution of white blood cell components: white blood cell count (WBCC), neutrophil count (NC), monocyte count (MC), and lymphocyte count (LC) in LDLR and NLDLR groups. (B) Comparison of NLR values between the 2 groups. LDLR, low-density lipoprotein receptor; NLDLR, non-low-density lipoprotein receptor. *P < .05 vs NLDLR.

Figure 2 illustrates the innate immunity profile distribution of the study population stratified according to genotype. Although no significant differences were found in WBCC (6.79 ± 1.36 vs 6.58 ± 1.86) or LC (2.01 ± 0.89 vs 2.02 ± 0.97), higher levels of MC and NC were observed in the LDLR group compared with the NLDLR group (for MC: 0.77 ± 0.21 vs 0.46 ± 0.12; for NC: 4.39 ± 1.32 vs 3.85 ± 1.14; for both *P* < 0.05) (Panel A). Panel B showed that NLR was significantly higher in the LDLR group than in the NLDLR group (2.27 ± 0.86 vs 2.05 ± 0.68, *P* < .05).

**Figure 3:**
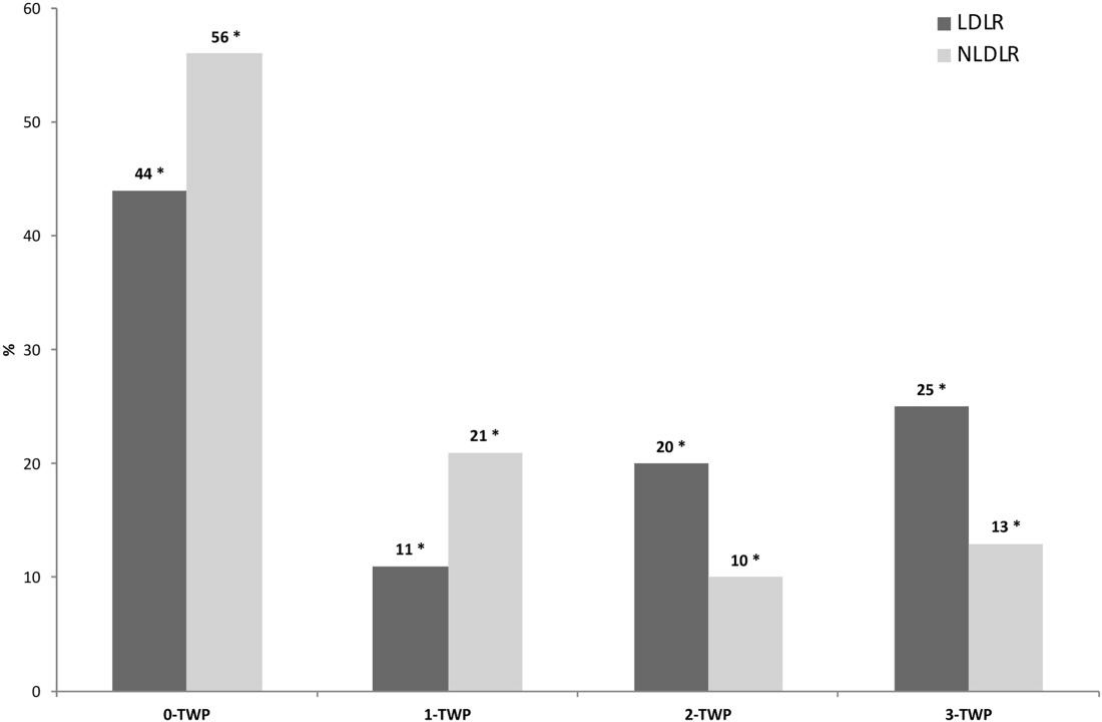
Atherosclerosis extension in FH subjects according to the number of vascular territories involved, stratified by LDLR mutation status. Subjects were stratified based on total vascular plaque involvement (TWP) into 0-TWP (no territory with plaque), 1-TWP (1 territory), 2-TWP (2 territories), and 3-TWP (3 territories). LDLR, low-density lipoprotein receptor; NLDLR, non-low-density lipoprotein receptor. *P < .05 vs NLDLR.

Figure 3 shows the extension of atherosclerosis 0-TWP, 1-TWP, 2-TWP, and 3-TWP in the 2 groups with a progressive increase in atherosclerotic burden according to LDLR mutation status (*P* for trend <.05). While subjects in the NLDLR group more frequently exhibited 0-TWP or 1-TWP compared to those in the LDLR group (for 0-TWP 56% vs 44%; for 1-TWP 21% vs 11%, for both *P* < .05), the prevalence of subjects with 2-TWP and 3-TWP was markedly higher in the LDLR group (for 2-TWP 20% vs 10%; for 3-TWP 25% vs 13%, for both *P* < .05).

Simple linear regression analysis showed that LDL-C (β = 0.250 ± 0.098, *P* < .05) and LDLR genotype (β = 0.205 ± 0.105, *P* < .05) were significantly associated with NLR, whereas no significant associations were observed for age, sex, BMI, systolic blood pressure, and smoking status (Table 3).

**Table 3:** Simple linear regression analyses evaluating neutrophil-to-lymphocyte ratio as dependent variable

Dependent variable	Neutrophil-to-lymphocyte ratio	
Independent variable	Coefficient β	*P* value
Age, years	0.004 ± 0.006	.39
Sex, male	0.002 ± 0.007	.11
BMI, kg/m^2^	0.012 ± 0.015	.39
LDL-C, mg/dL	0.250 ± 0.098	<.05
Systolic BP, mm Hg	0.004 ± 0.087	.17
Genotype, LDLR	0.205 ± 0.105	<.05
Smoking status	0.087 ± 0.103	.41

Abbreviations: BMI, body mass index; BP, blood pressure; LDL-C, low-density lipoprotein cholesterol.

Multivariate logistic regression analysis showed that age (*P* < .001), LDL-C (*P* < .001), smoking status (*P* < .05), and NLR (*P* < .05) were significantly associated with subclinical atherosclerosis (Table 4).

**Table 4:** Logistic regression of subclinical atherosclerosis in the study population

Dependent variable	Subclinical atherosclerosis	
Independent variable	Multivariate ORs (95% CIs)	
	Model	*P* value
Age, years	1.118 (1.085-1.153)	<.001
Gender, men = 1	1.121 (0.995-1.680)	.11
BMI, kg/m^2^	1.233 (1.022-1.996)	.12
LDL-C, mg/dL	1.189 (1.161-2.005)	<.001
Systolic BP, mm Hg	1.104 (1.083-1.729)	.15
Smoking status	1.122 (1.096-1.192)	<.05
NLR	1.119 (1.052-1.173)	<.05

Multivariate logistic regression model was used to estimate odds ratios (ORs) and 95% CIs, adjusted for age, gender, BMI, LDL-C, systolic blood pressure, smoking status, and NLR.

Abbreviations: BMI, body mass index; BP, blood pressure; LDL-C, low-density lipoprotein cholesterol; NLR, neutrophil-to-lymphocyte ratio.

## Discussion

In this study, we investigated the impact of genotype on immune-inflammatory status, assessed through NLR, and its relationship with SA in a cohort of FH subjects; to our knowledge, this is the first study exploring the relationship between NLR and SA in this population, with a specific focus on differences according to LDLR mutation status.

Our results showed that subjects with LDLR mutations exhibited a significantly higher NLR compared to those without LDLR mutations; moreover, it has been reported to be a significant association between SA and NLR. These findings support the hypothesis that vascular inflammation, influenced by genetic alterations in lipid metabolism, contributes to the pathogenesis of the atherosclerotic injury; in particular, prolonged exposure to elevated LDL-C levels in FH subjects may trigger innate immune activation (29).

The clinical significance of NLR as an inflammatory biomarker in the cardiovascular setting has been consistently demonstrated (30). A large systematic review and meta-analysis including >76 000 subjects showed that a high NLR was associated with an increased risk of coronary artery disease, stroke, and cardiovascular mortality (10). Moreover, García-Escobar et al showed that elevated NLR levels are independently associated with adverse cardiovascular outcomes, even in the absence of traditional risk factors or acute coronary syndromes in a cohort of patients with heart failure, established ASCVD, and high-risk individuals with subclinical atherosclerosis (31). Furthermore, Adamstein et al found that NLR played a key role in the interaction between systemic inflammation and endothelial dysfunction in a cohort of patients with established ASCVD enrolled in large randomized clinical trials (CANTOS, JUPITER, SPIRE-1, SPIRE-2, CIRT) (32). These findings reinforce the concept of atherosclerosis as a chronic inflammatory condition and support the adoption of NLR as a simple, reproducible, and cost-effective biomarker for detecting an early immune activation (33).

The higher NLR observed in the LDLR group reflected a selective expansion of neutrophil and monocyte counts without significant alterations in lymphocyte levels or total WBCC. This finding suggests a specific activation of the myeloid compartment, in line with Bosco et al, who demonstrated that in FH subjects with subclinical atherosclerosis the immune profile could be stimulated by prolonged exposure to elevated LDL-C levels (34)

This mechanistic interpretation is further supported by the model proposed by Libby et al who highlighted the central role of the NOD-like receptor family pyrin domain containing 3, IL-1β, IL-6 axis in the cross talk between cholesterol accumulation and leukocyte recruitment with an increased inflammatory cytokine production (35). These findings are consistent with the current concept of atherosclerosis as a chronic inflammatory disease, in which cumulative LDL-C exposure sustains vascular damage through persistent activation of the innate immune system (36). In this context, the elevated NLR observed in LDLR group may reflect a subclinical inflammatory state and thus it could be useful to identify FH subjects with a more pronounced atherosclerotic injury.

The combination of elevated LDL-C levels and systemic inflammation represents a key determinant of atherosclerosis progression (37) and thus subjects with a worse lipo-inflammatory status may have a higher cardiovascular risk (38).

In this context, our study showed that FH subjects carrying LDLR mutations presented a more adverse lipo-inflammatory profile characterized by elevated LDL-C levels and higher NLR; thus, the presence of both alterations may promote the atherosclerotic injury in these subjects.

Moreover, the chronic accumulation of LDL-C within the arterial wall promotes endothelial dysfunction and activates pattern recognition receptors such as Toll-like receptor 4 leading to the release of pro-inflammatory chemokines including monocyte chemoattractant protein-1 or IL-8, which mediate the recruitment and activation of neutrophils and monocytes (39).

Autoimmune thyroid disease may influence both LDL levels and leukocyte distribution, with hypothyroidism often presenting with hypercholesterolemia and relative lymphocytosis (40). This interaction is well recognized, and we excluded subjects with clinical or biochemical evidence of thyroid involvement at baseline to avoid secondary effects on lipid and inflammatory parameters.

Hormonal disorders and systemic treatments can influence both lipid metabolism and inflammatory indices. For this reason, subjects with endocrine diseases or receiving hormonal therapies were excluded to avoid secondary effects unrelated to FH. Recent data showed that testosterone therapy has been associated with variations in LDL-C cholesterol and total cholesterol (41). These observations reinforce the importance of excluding hormonal interference when evaluating inflammatory or lipid-related markers in FH subjects. Neutrophils contribute to vascular damage through the release of proteolytic enzymes such as elastase and myeloperoxidase, reactive oxygen species, and neutrophil extracellular traps (NETs) that exacerbate endothelial injury and promote plaque instability (42).

NET-related activation may also contribute to the relative increase in circulating neutrophils observed in LDLR mutation carriers, as cholesterol-induced NET formation can enhance peripheral neutrophil recruitment and persistence (43). Moreover, in the subendothelial space monocytes differentiate into pro-inflammatory M1 macrophages that sustain foam cell formation and atherosclerosis progression (44). An increased lipo-inflammatory activation may underlie the selective expansion of neutrophil and monocyte populations, as reflected by the higher NLR values in LDLR mutation carriers.

CRP remained within the normal range in our population. This finding is consistent with evidence indicating that early vascular inflammation and endothelial activation may occur in the absence of systemic inflammatory elevation. Recent data in untreated heterozygous FH have shown increased endothelial inflammatory activity despite normal CRP levels, suggesting that local arterial inflammation can precede systemic activation and that cellular indices such as NLR may be more sensitive than CRP in this context (45).

As concerns the vascular profile, we observed a different distribution of subclinical atherosclerosis according to LDLR mutation status. Subjects with an LDLR mutation showed a higher prevalence of atherosclerosis extension (TWP ≥2), suggesting a more severe vascular injury. Our findings are in line with the hypothesis that impaired LDLR function leads to sustained exposure to markedly elevated LDL-C levels, resulting in a more pronounced cholesterol deposition and progressive vascular injury (46). As reported by Besseling et al, cumulative LDL-C exposure plays a key role in determining the severity and extent of atherosclerosis in FH, with mutation carriers showing earlier onset and more aggressive atherosclerotic disease (47). In this context, in FH subjects with an LDLR higher LDL-C levels as well as an increased NLR may promote the progression of atherosclerotic injury in several districts.

Although our cohort was evaluated without lipid-lowering therapy, recent evidence shows that intensive LDL-C reduction reduces atherosclerotic burden. High-intensity statins and PCSK9 inhibitors have consistently demonstrated meaningful effects on plaque burden and on the progression of subclinical atherosclerosis (48).

There are several limitations to our study. First, this was a cross-sectional study and thus it was not possible to evaluate a causal association between elevated NLR and vascular injury. It is important to highlight that both LDL-C levels and LDLR mutation status were independently associated with NLR in our regression model. Therefore, although genotype-related alterations in lipid metabolism may contribute to immune activation, the cross-sectional design does not allow us to the effect of genetic background from that of cumulative LDL-C exposure. The study did not include additional inflammatory biomarkers or cytokine profiling and thus it was not possible to assess the contribution of specific immune mediators or to characterize the activation status of distinct leukocyte subpopulations such as classical vs nonclassical monocytes or neutrophil subsets. Although NLR was measured under stable clinical conditions, a single measurement may not fully reflect intraindividual variability. NLR is influenced by infections, autoimmune diseases, stress, and corticosteroid exposure; however, subjects with these conditions were excluded. Therefore, longitudinal studies with repeated measurements are needed to better characterize its temporal stability in FH subjects.

Moreover, our population did not undergo CT angiography, despite its ability to provide detailed plaque characterization and identify vulnerable lesions (49). However, we adopted less invasive and widely available diagnostic tools, including the Agatston CAC score, a validated marker of subclinical atherosclerosis and a strong predictor of cardiovascular events in both the general population and FH subjects (50). Finally, the cardiovascular prognostic role of NLR in FH, as well as its modulation in response to pharmacological interventions, remains to be determined. Further prospective longitudinal studies are needed to evaluate the role of NLR in the cardiovascular risk stratification and therapeutic monitoring in FH subjects.

## Conclusions

In conclusion, our findings suggest that FH subjects with LDLR mutations had a higher NLR and a more severe atherosclerosis distribution. Our findings support the role of NLR as a noninvasive biomarker of early immune activation and highlights the importance of lipo-inflammatory status evaluation in FH subjects. Further prospective longitudinal studies are needed to evaluate the role of NLR in the cardiovascular risk stratification and therapeutic monitoring in FH subjects.

## Data Availability

The data underlying this article will be shared on reasonable request to the corresponding author.

## Abbreviations

ApoB: apolipoprotein B
ARH: autosomal recessive hypercholesterolemia
ASCVD: atherosclerotic cardiovascular disease
BMI: body mass index
CAC: coronary artery calcium
CT: computed tomography
FH: familial hypercholesterolemia
HDL-C: high-density lipoprotein cholesterol
LDL-C: low-density lipoprotein cholesterol
LDLR: low-density lipoprotein receptor
LC: lymphocyte count
NC: neutrophil count
NETs: neutrophil extracellular traps
NLDLR: non-low-density lipoprotein receptor
NLR: neutrophil-to-lymphocyte ratio
PCSK9: proprotein convertase subtilisin/kexin type 9
SA: subclinical atherosclerosis
TG: triglycerides
WBCC: white blood cell count
TWP: territorial vascular plaque involvement
